# Investigation of Correlates of Protection Against Gonococcal Infection by Comparative Immunoprofiling of Responses in Experimental and Clinical Studies

**DOI:** 10.1093/infdis/jiag216

**Published:** 2026-04-29

**Authors:** Samantha A McKeand, Fidel Ramirez Bencomo, Anthony Soc, Rebekah A Jones, Angela Thistlethwaite, Zhenyi Gu, Eunice Nduati, Eduard J Sanders, Ann E Jerse, Jeremy P Derrick, Christoph M Tang

**Affiliations:** Sir William Dunn School of Pathology, University of Oxford, Oxford, United Kingdom; Lydia Becker Institute of Immunology and Inflammation, School of Biological Sciences, Faculty of Biology, Medicine and Health, The University of Manchester, Manchester, United Kingdom; Henry M. Jackson Foundation for the Advancement of Military Medicine, Bethesda Maryland, USA; Sir William Dunn School of Pathology, University of Oxford, Oxford, United Kingdom; Lydia Becker Institute of Immunology and Inflammation, School of Biological Sciences, Faculty of Biology, Medicine and Health, The University of Manchester, Manchester, United Kingdom; Sir William Dunn School of Pathology, University of Oxford, Oxford, United Kingdom; KEMRI-Wellcome Trust Research Programme, Hospital Road, Kilifi, Kenya; The Aurum Institute, Johannesburg, Gauteng, Republic of South Africa; Department of Microbiology and Immunology, Uniformed Services University, Bethesda, Maryland, USA; Lydia Becker Institute of Immunology and Inflammation, School of Biological Sciences, Faculty of Biology, Medicine and Health, The University of Manchester, Manchester, United Kingdom; Sir William Dunn School of Pathology, University of Oxford, Oxford, United Kingdom

**Keywords:** *Neisseria gonorrhoeae*, Bexsero, OMVs, correlates of protection, systems serology

## Abstract

**Background:**

*Neisseria gonorrhoeae* is a major sexually transmitted pathogen with rising antimicrobial resistance. Epidemiological studies suggest meningococcal outer membrane vesicle (OMV) vaccines, eg 4CMenB (Bexsero), offer partial cross-protection, but immune correlates remain undefined.

**Methods:**

Using a murine genital tract infection model, we compared systemic and mucosal responses after immunization with Bexsero, gonococcal OMVs (*Ng* OMVs) with alum, or alum alone. Vaccinated mice were challenged intravaginally with *N. gonorrhoeae*. Serum bactericidal activity (SBA) was measured, and antigen-specific IgG responses were profiled using gonococcal protein microarrays. Murine responses were compared with high-risk Bexsero-immunized humans and patients with gonococcal infection.

**Results:**

Bexsero significantly accelerated bacterial clearance compared with *Ng* OMV or alum. Serum and vaginal IgG profiles were highly correlated, revealing distinct antigenic signatures between groups. Bexsero elicited responses to gonococcal orthologs of its recombinant antigens (GNA1030, GNA2091, NHBA), whereas *Ng* OMVs induced Opa, PilQ, and MtrE responses. SBA did not correlate with clearance; however, early PMN influx was associated with reduced bacterial load. Antigen-specific IgG patterns were concordant between Bexsero-immunized mice and humans.

**Conclusions:**

Protein microarrays with multivariate analysis identified antibody signatures associated with protection, supporting the translational value of this model for defining correlates of protection and guiding gonococcal vaccine development.

## Teaser

Meningococcal OMV vaccination confers cross-protection against gonococcal infection, and induces systemic and mucosal IgG signatures, with responses in mice reflecting those in human vaccinees. Protection in mice was associated with early PMN influx in the genital tract following infection.

Gonorrhea is a sexually transmitted infection caused by *Neisseria gonorrhoeae* (*Ng*), with over 80 million cases annually [1]. Asymptomatic infection in women increases the risk of complications, including infertility, ectopic pregnancy [2], low birth weight [3] and neonatal blindness [4]. Emergence of multidrug-resistance has rendered many antibiotics ineffective [5]. Due to the threat of untreatable infection, effective vaccines are urgently needed to mitigate the impact of gonorrhea on reproductive health.

Recent epidemiological evidence indicates that outer membrane vesicle (OMV)-based vaccines derived from *Neisseria meningitidis* may offer some cross-protection against gonorrhea [6], with approximately 30% reduction of symptomatic gonorrhea among vaccinees versus unvaccinated individuals across multiple settings [6–8]. Evidence comes from use of MeNZB vaccine [9] and 4CMenB (Bexsero) [10] which contain the same OMVs; Bexsero has additional recombinant factor H binding protein (fHbp), NadA, and NHBA. NadA is not expressed by *Ng*, fHbp is not on the gonococcal surface [11], and NHBA exhibits limited immunogenicity [12, 13].

These clinical observations have been replicated in the murine genital tract infection model [14–16]. Conversely, OMVs from *Ng* confer minimal or no protection [17], unless combined with immunomodulators, which probably overcome gonococcal immune evasion mechanisms that prevent development of protective immunity during natural infection [18–20].

Despite these advances, immunological correlates of protection remain unclear [17, 21]. However, the murine gonococcal genital infection model could help identify relevant correlates [15, 16]. Zhu *et al* [16] compared cross-protection induced by Bexsero and OMVs from engineered *N. meningitidis* (MC58ΔABR) versus adjuvant-only. Cytokine assays showed elevated splenocyte responses post-challenge to gonococcal OMVs while potential immunological correlates differed between vaccines, implying distinct protective mechanisms. Zeppa *et al* [15] grouped mice given Bexsero as “protected” or “non-protected”. Post-challenge analyses revealed strong mucosal Th1-skewed immunity, with protection linked to elevated tissue-resident T cells and cytokines in the genital tract, suggesting site-specific immunity.

Here, we sought to identify correlates of protection against gonococcal infection but using distinct approaches. We compared serological and mucosal Immunoglobulin G (IgG) responses in mice immunized with Bexsero or OMVs from *Ng* (*Ng* OMVs) with alum, similar to Bexsero. We reasoned that including responses to *Ng* OMVs, which were not expected to protect, would help discriminate protective from non-protective signatures. We employed protein microarrays containing 91 gonococcal antigens [12] to profile IgG responses, and compared murine responses to those in individuals following Bexsero immunization or gonococcal infection. Results emphasize the translational value of the murine model and indicate that profiling of responses against individual antigens could allow machine learning to identify correlates of protection.

## METHODS

### Ethical Approval

Fifty participants (aged 18–25), comprising sex workers and/or men-who-have-sex-with-men were immunized with Bexsero at Kenya Medical Research Institute (KEMRI) [12]. Ethical approval was granted by the KEMRI Scientific and Ethical Review Unit (CSC 182) and University of Oxford (16–20). Animal experiments were conducted according to protocols approved by the Uniformed Services University's Institutional Animal Care and Use Committee.

### Generation of Outer Membrane Vesicles

*Ng* FA1090 lacking *rmpM* (FA1090Δ*rmpM*) [22] was grown overnight on gonococcal base medium (GCB) plates at 37 °C in 5% CO_2_, resuspended in 50 mL liquid GCB containing 1% Vitox at an optical density (OD_600_) of .1, then incubated at 37 °C in 5% CO_2_ with shaking at 180 rpm until the OD_600_ reached 1. Bacteria were pelleted and supernatants filtered (0.22 μm), and OMVs obtained *via* ultracentrifugation at 235 000*×g* for 2 hours at 4 °C, washed with PBS and ultracentrifuged again.

### Animal Immunizations and Challenge

Female BALB/c mice (4 weeks old) (Charles River, #555NCIBALB/C) were randomized and immunized subcutaneously with *Ng* OMV (12.5 µg/dose) with 2% alhydrogel (“alum,” InvivoGen), 250 µL Bexsero (Glaxo-Smith-Kline), or alum alone (adjuvant control) on days 0, 21, and 35. Serum and vaginal lavages were collected 10 days after the final immunization (d45). Vaginal washes (VW) consisted of three 35 µL PBS douches/vaginal cavity, then centrifuged at 2000*×g*. Three weeks post-immunization, mice in anestrus or the diestrus stage were implanted subcutaneously with 6.5 mg slow-release 17β-estradiol (Innovative Research of America) and treated with antibiotics [23]. Two days later, mice were challenged vaginally with 10^6^ CFU of *Ng* F62 in 20 µL of saline. Vaginal swabs were cultured on GCB plates with VCNT using a Spiral Plater (Interscience).

Clearance was measured as percentage of culture-positive mice post-challenge and analyzed by the Log-Rank test. CFU/mL recovered from each group was compared by 2-way analysis of variance (ANOVA) with Tukey's adjustment. Kruskal-Wallis's test with Dunn's multiple comparison test was employed to assess the total area under the curve (AUC) over 7 days.

Vaginal swabs were used to prepare slides to evaluate polymorphonuclear leukocyte (PMN) influx, and PMNs were quantified as the percentage of 100 counted cells. Associations between PMN counts and bacterial burden were assessed using Hotelling's *T*^2^ test. Pairwise comparisons were performed with Bonferroni adjustment for multiple testing in R (v4.3.1). Ellipses represent the 95% confidence regions for each group based on a multivariate *t*-distribution and illustrate the distribution and separation of the data.

### Serum Bactericidal Assays

*Ng* F62 was resuspended in PBS to 6 × 10^3^ CFU/mL then incubated with dilutions of heat-inactivated sera in Eagle's Minimal Essential Medium for 10 minutes at 37 °C with 5% CO_2_. IgG/IgM-depleted human sera (Pel-Freez, 3% final concentration) was added and incubated at 37 °C with 5% CO_2_ for 45 minutes. Bacterial survival was expressed relative to complement-only controls. For sialylation, F62 was grown in liquid GCB with 2 μg/mL cytidine-5′-monophospho-*N*-acetylneuraminic acid. Results were compared with Dunn's multiple comparison.

### Gonococcal Protein Microarrays

Custom microarrays (Arrayjet Limited, UK) contained 91 distinct proteins from *N. gonorrhoeae* FA1090 [12] with JetStar printing buffer (Arrayjet, UK). Sera were diluted 1:300 with Tris-buffered saline (TBS), which provided the best signal-to-noise ratio [22]. 50 µl were added to each array and incubated at 20 °C for 1 hour. After washing 3 times with 300 μL TBS-T (0.05% Tween-20) and once in TBS for 10 minutes, slides were incubated for 1 hour at 20 °C with 50 μL of a goat antimouse IgG Fc (DyLight® 650) (ab97018, Abcam, UK). VW were diluted 1:5 with TBS.

### Computational Methods

Principal component analysis (PCA) plots were calculated using factoextra in R (v1.0.7). For eXtreme Gradient Boost (XGBoost) analysis, SBA was calculated as a percentage (complement-only control CFU - experimental CFU/complement-only control CFU*100) from a 1:2000 murine serum dilution, as SBAs at this dilution with sera from individual mice gave a range of values. Relative SBAs were used for regression of the serum IgG antigen reactivity against antigens from *Ng* FA1090 employing XGBoost in R from the caret package [24]. The top 10 antigens were used to construct a simplified linear model. F-statistic and *P* value were then calculated from this model. To examine correlations with vaginal CFU counts, CFU counts were summed from day 3 to 7 inclusive to calculate the AUC. The same process of XGBoost fitting was employed as for SBA analysis.

## RESULTS

### Mice Immunized With Bexsero, but Not *Ng* Outer Membrane Vesicles, are Protected From Gonococcal Challenge

**Figure 1.**
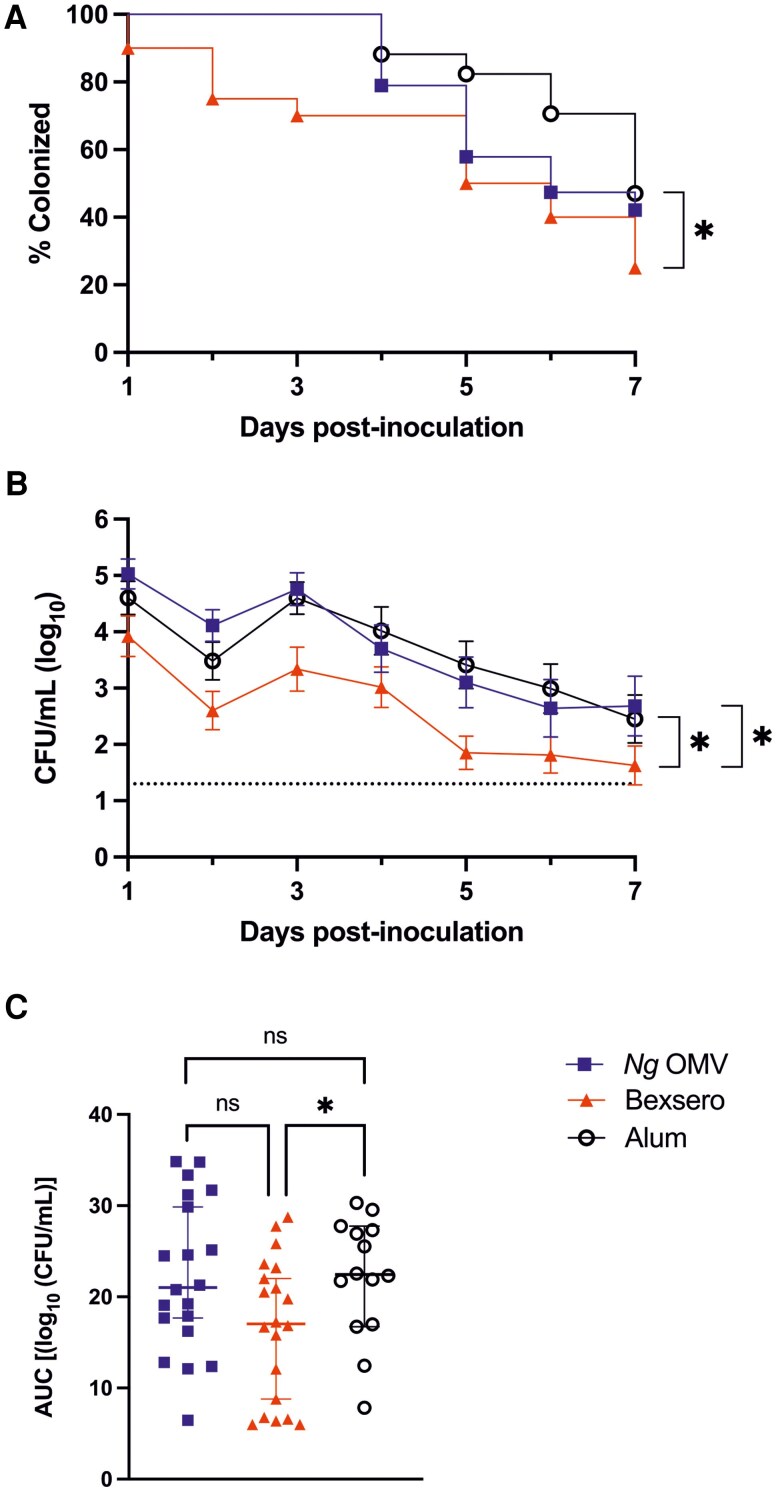
Mice immunized with Bexsero but not with *Ng* outer membrane vesicle (OMV) or alum are protected against challenge with *N. gonorrhoeae*. *A*, Percentage of mice in each group colonized with *N. gonorrhoeae* F62 after inoculation on day 0 with 10^6^ CFU intravaginally, and (*B*) bacterial burden (in CFU/mL) in vaginal washes (VW) on days post-challenge. *C*, The AUC of bacterial recovery over the time course of infection. Each mouse is shown as an individual point; n = 20, 19, and 14 for the *Ng* OMV, Bexsero, and alum-only groups, respectively. *P* < .05 (*), not significant (ns) for comparison between groups.

Following immunization with Bexsero or *Ng* FA1090 OMV with alum, using equivalent amounts of OMVs, mice were challenged intravaginally with *Ng* F62; control mice were immunized with alum. As expected, immunization with *Ng* OMVs did not protect, as assessed by clearance (Figure 1*A*), while Bexsero vaccination significantly accelerated *Ng* clearance, with the percentage of *Ng*-infected mice showing a stepwise decrease from day 1 post-challenge onwards. There was a significant difference in the average number of CFU/mL over time (Figure 1*B*) as well as in the total bacterial burden (AUC) (Figure 1*C*) in Bexsero-vaccinated mice compared with other groups.

### Local Mucosal IgG Responses Against Outer Membrane Vesicle Antigens Mirror Those in Sera

**Figure 2.**
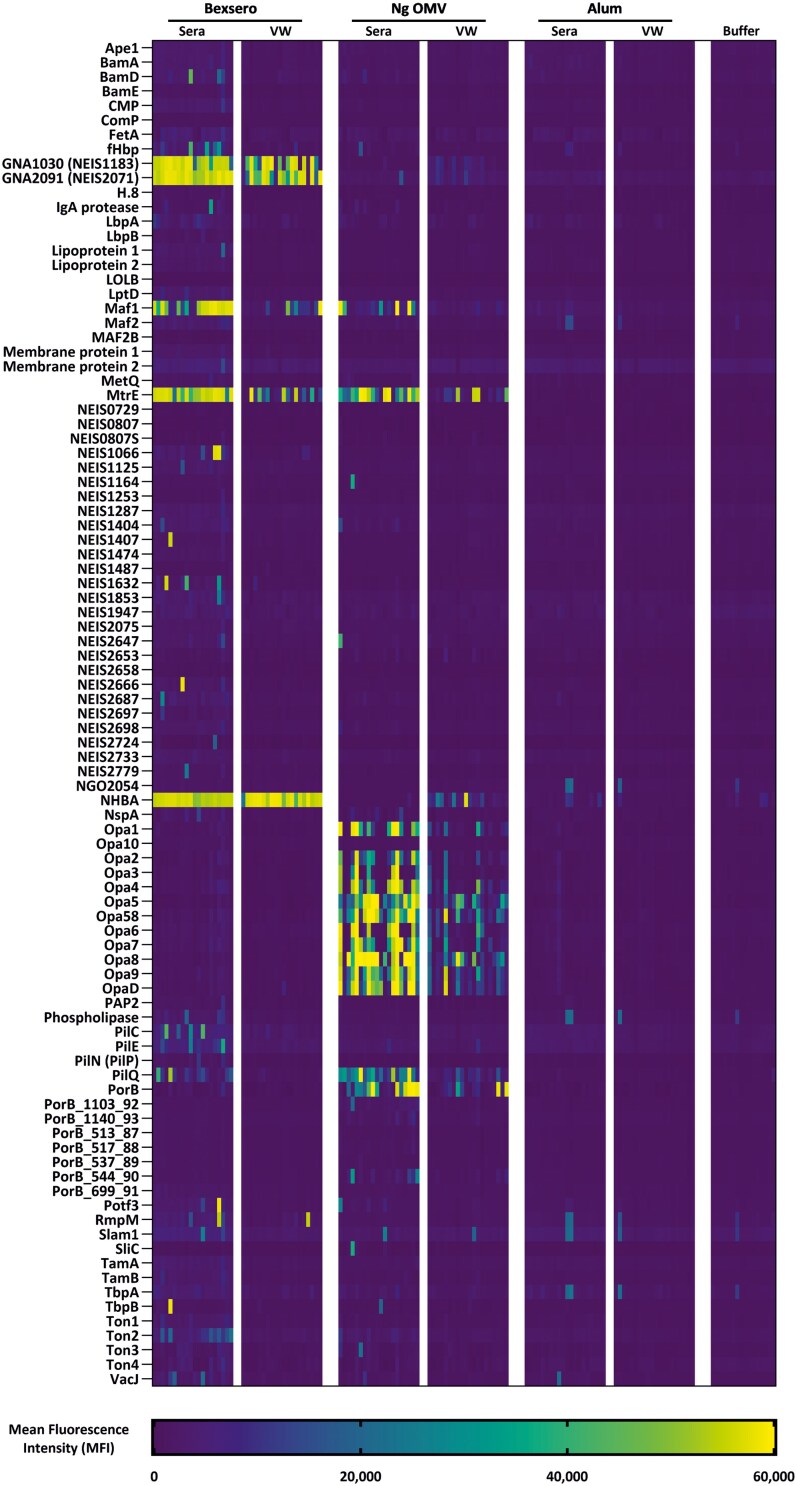
Heatmap of serum and vaginal antigen-specific IgG responses from mice immunized with Bexsero, *Ng* outer membrane vesicles (OMVs), or adjuvant alone. Each column represents IgG responses, measured by mean fluorescence intensity (MFI), derived from a single mouse and separated into the experimental groups, Bexsero, *Ng* OMV and alum. Each group is further subdivided into IgG responses derived from serum or vaginal washes (VW) (the column order is preserved for each animal). The color range uses the viridis scale (right key) to show the MFI. Alternative antigen nomenclature is detailed in Supplementary Table 1.

To define correlates of protection, systemic and mucosal IgG were analyzed at d45 (ie 2 wk pre-challenge) using gonococcal antigen microarrays [12, 22] containing proteins from *Ng* FA1090. As IgA signals from VWs were low, this was not pursued. Alum-only mice showed low levels of IgG comparable to buffer controls (Figure 2), whereas *Ng* OMV-immunized mice demonstrated strong reactivity against MtrE, the outer membrane component of a drug transporter [25], type IV pilus protein PilQ [26], and Opa (opacity) proteins [27]. Results for mice receiving Bexsero are markedly distinct, with higher responses detected against the gonococcal orthologs of the recombinant antigen NHBA, and carrier proteins GNA1030 and GNA2091. However, there were significant differences with other antigens, with lower responses against Opas and elevated responses against MtrE and Maf1 (Figure 2). Results from serum and VW samples were similar. To examine this quantitatively, we plotted responses in serum and VW for each antigen and conducted linear regression analysis (Supplementary Figure 1), identifying a linear relationship (*P* < 2.2 × 10^−16^) between serum and mucosal IgG responses, indicating that serological IgG responses mirror those in the genital tract.

### Mice Immunized With Bexsero Show Distinct Responses From Those Immunized With *Ng* Outer Membrane Vesicles

**Figure 3.**
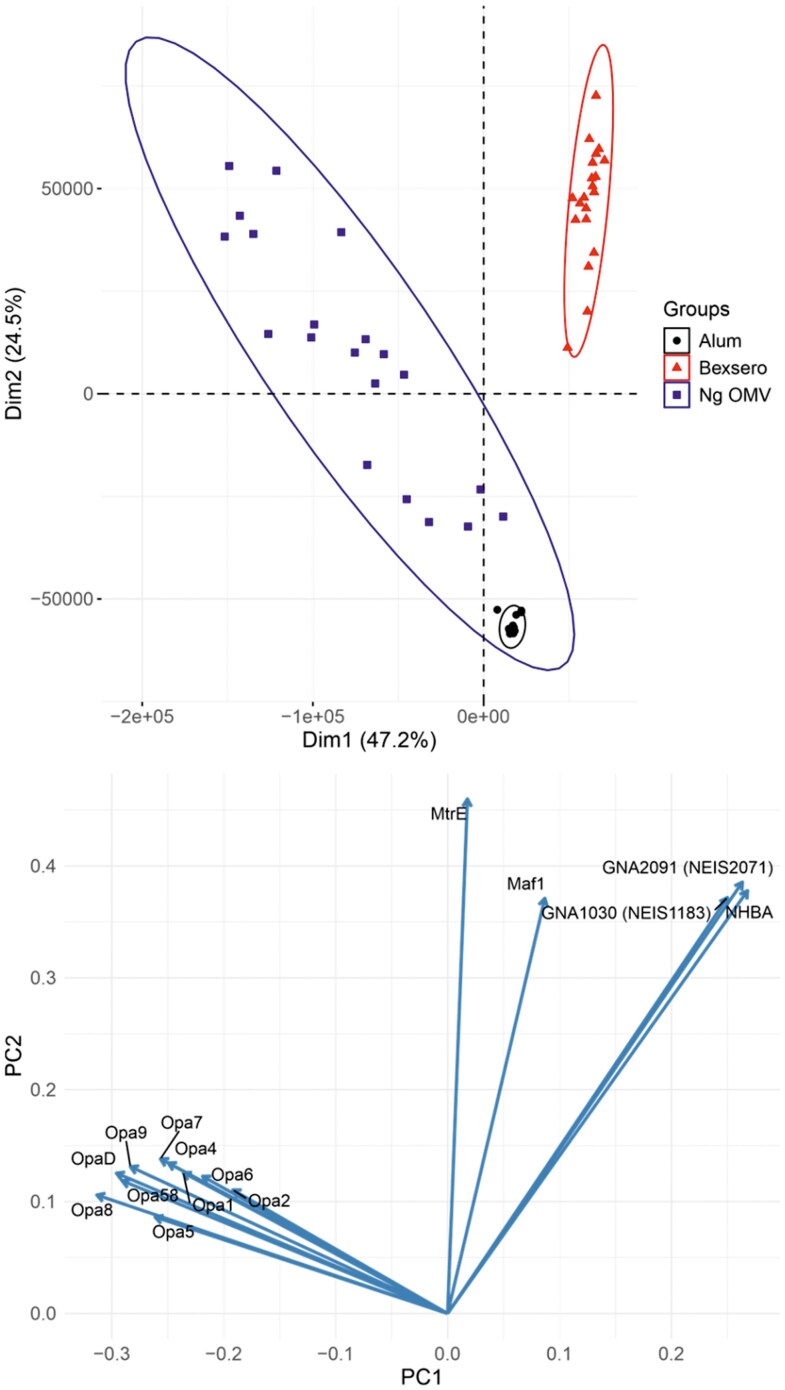
Principal component analysis (PCA) of serum IgG responses to Bexsero or *Ng* outer membrane vesicle (OMV). Top panel, PCA plot with points colored by experimental group as shown. Ellipses are plotted at a 95% confidence level (this proportion of data points is expected to fall within the ellipse if the data are normally distributed). Bottom panel, vector plot showing contributions of individual antigens to PC1 and PC2. For clarity, only the top 15 contributing antigens are shown.

To identify differences between vaccination groups, PCA was applied to the entire dataset; 73% of the total variance was captured in PC1 and PC2 (Figure 3, upper panel). There is clear separation between serum samples from the Bexsero and *Ng* OMV groups. Most of the separation between these groups lies along PC1, which is dominated by Opa proteins which have highly divergent extracellular loops [28]. Given the divergence in sequence between Opa proteins in Bexsero and *Ng* FA1090 (on microarrays) [12], it is understandable that anti-Opa responses are comparatively strong in the *Ng* OMV group (Figure 2). Separation in PC2 is driven by reactivities against gonococcal homologs of the recombinant antigens in Bexsero along with Maf1 and MtrE. Analyses were repeated omitting the recombinant antigens (Supplementary Figure 2), and the conclusions are similar to those in Figure 3, with separation largely driven by anti-Opa responses, and PilQ.

**Figure 4.**
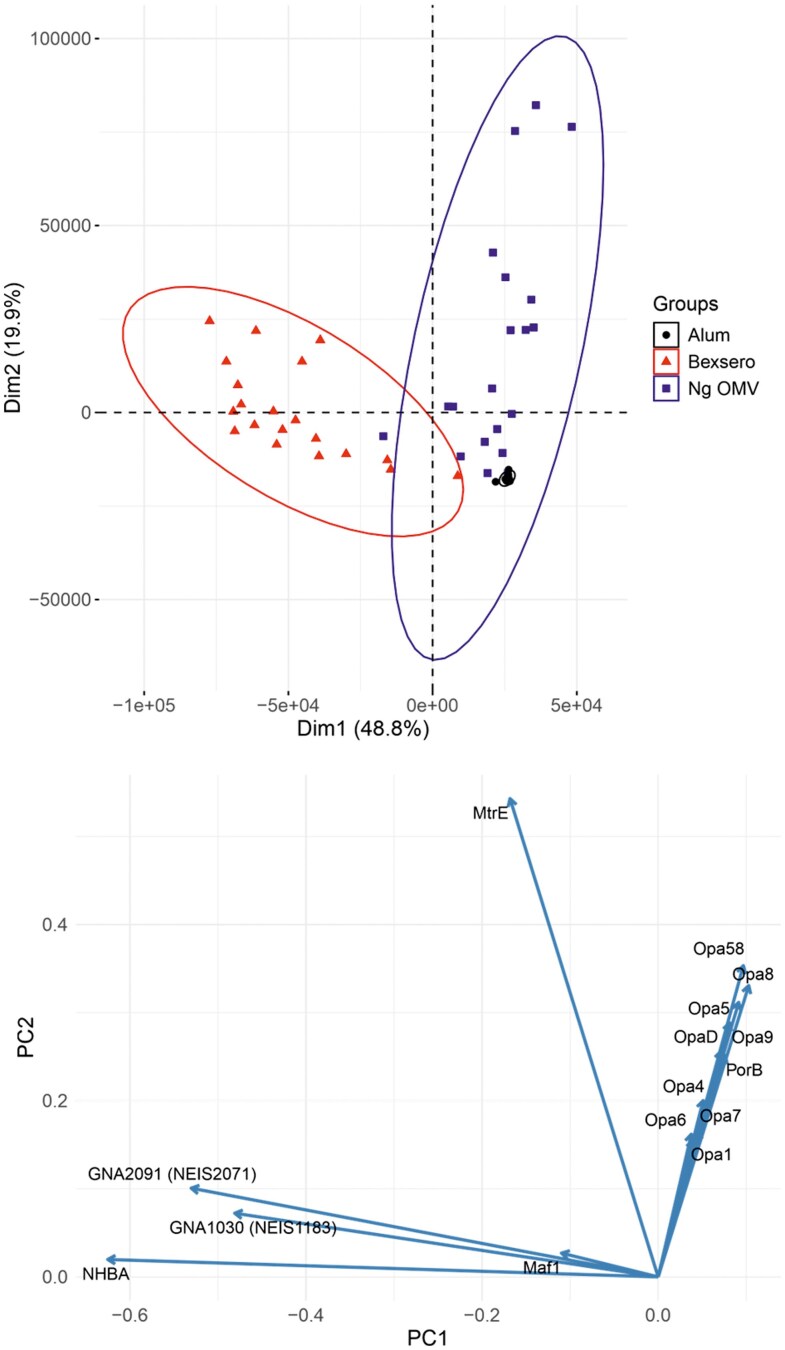
Principal component analysis (PCA) of IgG responses in vaginal washes post-immunization with Bexsero, *Ng* outer membrane vesicles (OMVs) or adjuvant. Top panel, PCA plot with points colored by experimental group. Ellipses are plotted at a 95% confidence level. Bottom panel, vector plot, showing contributions of individual antigens (indicated) to PC1 and PC2. For clarity, only the top 15 contributing antigens are shown.

PCA of antibody reactivities from the VW samples showed that, similar to serum IgG, the Bexsero and *Ng* OMV groups were well separated along the PC1 axis; however, this was driven by the responses against recombinant antigens, unlike results with serum (Figure 4). Anti-Opa reactivities drive dispersion in PC2. Omission of responses against the recombinant antigens revealed a more complex picture (Supplementary Figure 3); dispersion of the *Ng* OMV data points was very wide. Differentiation from the Bexsero group is driven by contributions from multiple antigens, including Opas, PilQ, and PorB.

### Murine Responses to Bexsero Correlate With Those Seen in Human Vaccinees

**Figure 5.**
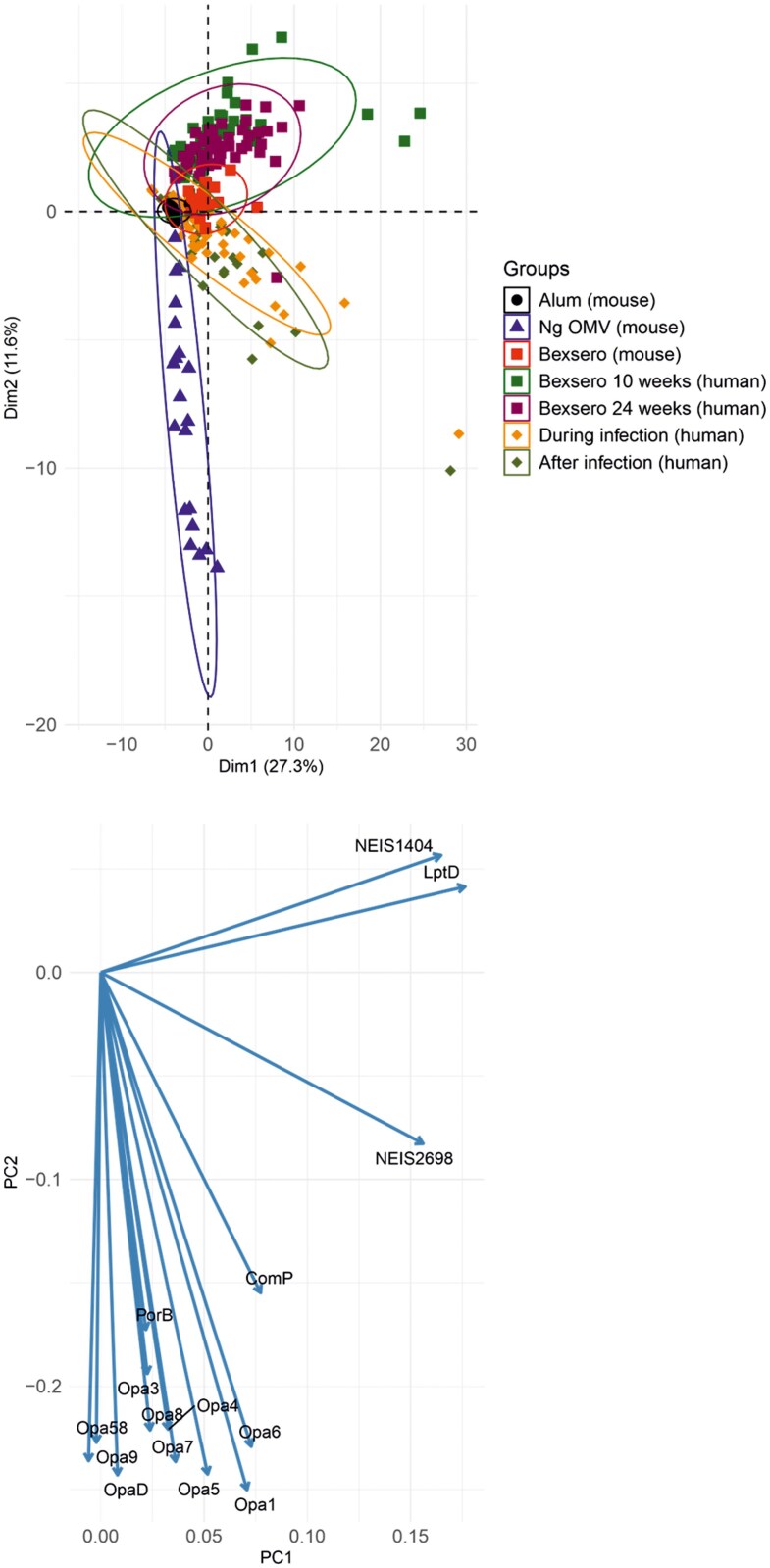
Principal component analysis (PCA) comparison of serum IgG responses between mouse and human clinical samples. Top panel, PCA plot (with each point representing an individual animal or individual) colored by experimental group. Ellipses are plotted at a 95% confidence level. The human data are from Stejskal *et al* [12]. Bottom panel, vector plot, showing contributions of individual antigens to PC1 and PC2. Data were scaled before calculation. For clarity, only the top 15 contributing antigens are shown.

Combining immunoprofiles from multiple microarray datasets can be used to compare antigen reactivity profiles from different datasets. We therefore compared responses induced in mice with high-risk individuals immunized with Bexsero, and patients during/after gonococcal infection [12] (Figure 5). The murine IgG responses following Bexsero immunization (this study) overlap significantly with those seen post-immunization of human volunteers at both the 10- and 24-week time points [12]. Data from Bexsero-immunized mice and humans are well separated from the murine responses to *Ng* OMVs. Interestingly, individuals with gonococcal infection fall between these clusters, likely reflecting strain diversity in natural infection compared with FA1090-derived OMVs. The top 15 antigens responsible for differentiation between these clusters (Figure 5, lower panel) show the influence of the Opa proteins driving separation along PC2. Thus, a complex combination of antigens drives separation between groups.

### SBAs From Immunized Mice Do Not Correlate With Outcome

**Figure 6.**
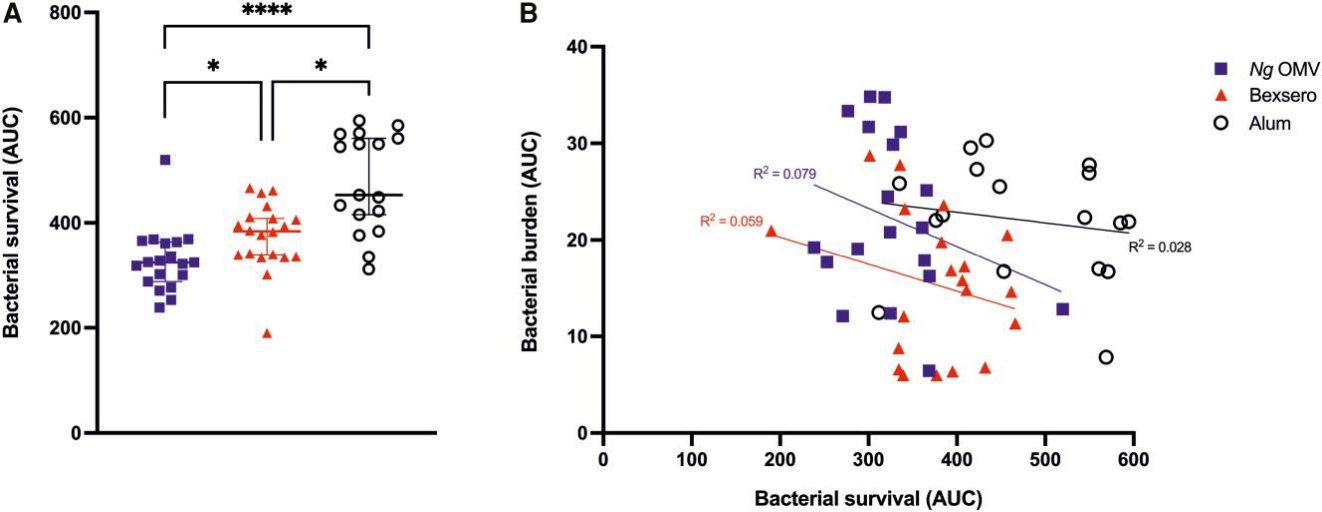
Correlation between pre-challenge serum bactericidal activity (SBA) and bacterial burden in individual mice during challenge. SBAs with sera from mice immunized with *Ng* outer membrane vesicles (OMVs), Bexsero, or alum alone 10 d after the final immunization (ie pre-challenge). *A*, *N. gonorrhoeae* F62 was incubated with serial dilutions of heat-inactivated serum from individual mice with IgG/IgM-depleted human serum as the complement source. Percentage survival was calculated as the difference between the number of colonies recovered from the complement-only controls. The AUC for bactericidal activity across serum dilutions from each mouse was calculated and compared across immunization groups (Dunn's multiple comparisons; *P* < .05 (*), *P* < .0001 (****). *B*, AUC derived from SBA was plotted with the AUC of the corresponding bacterial burden (in CFU/mL). Each point represents data from an individual mouse.

To evaluate antibody functionality, sera from mice vaccinated with Bexsero, *Ng* OMVs, or alum collected on d45 were tested in SBAs against the challenge *Ng* strain F62, allowing comparison of SBAs with clearance in vivo without the complication of different strains being used in assays. The AUC (Figure 6*A*) and SBA_50_ titer (Supplementary Figure 4) were calculated for each mouse from serum bactericidal activity across serum dilutions and compared between immunization groups (Supplementary Figure 5 for all SBA data). Both Bexsero and *Ng* OMV groups exhibited statistically significant increases in SBA compared with the alum group (*P* = .0430 and *P* < .0001, respectively), with *Ng* OMV inducing greater overall bactericidal activity compared with Bexsero (*P* = .0334). When the assay was repeated with sialylated bacteria, no bacterial killing was observed in any immunization groups (Supplementary Figure 6).

To investigate whether SBA correlated with accelerated clearance, SBA AUC values for each mouse were correlated with the bacterial burden following gonococcal challenge (Figure 6*B*). Overall, an inverse correlation was observed between burden and SBA, suggesting that SBA is not a reliable correlate of protection.

### Correlation of Antibody Reactivity Data With Serum Bactericidal Activity

To correlate antibody profiles with SBA, XGBoost was applied to all groups. The top 10 predictive antigens were then used to fit a simplified linear model to SBA values, which provided an excellent fit to the data (*P* = 1.035 × 10^−6^; adjusted *R*^2^ = 0.51). PorB was consistently the most influential antigen, followed by PilQ and BAM complex members, BamD and BamE. When XGBoost was applied to correlate pre-challenge IgG profiles with bacterial burden, the fit was much weaker (*P* = .023, adjusted *R*^2^ = 0.213), consistent with the limited association between SBA and accelerated clearance. Nonetheless, this approach could be used to identify antigen combinations associated with protection, if sufficient statistical power could be obtained.

### Bexsero Vaccination Induces an Early Neutrophil-associated Shift in Bacterial Clearance

**Figure 7.**
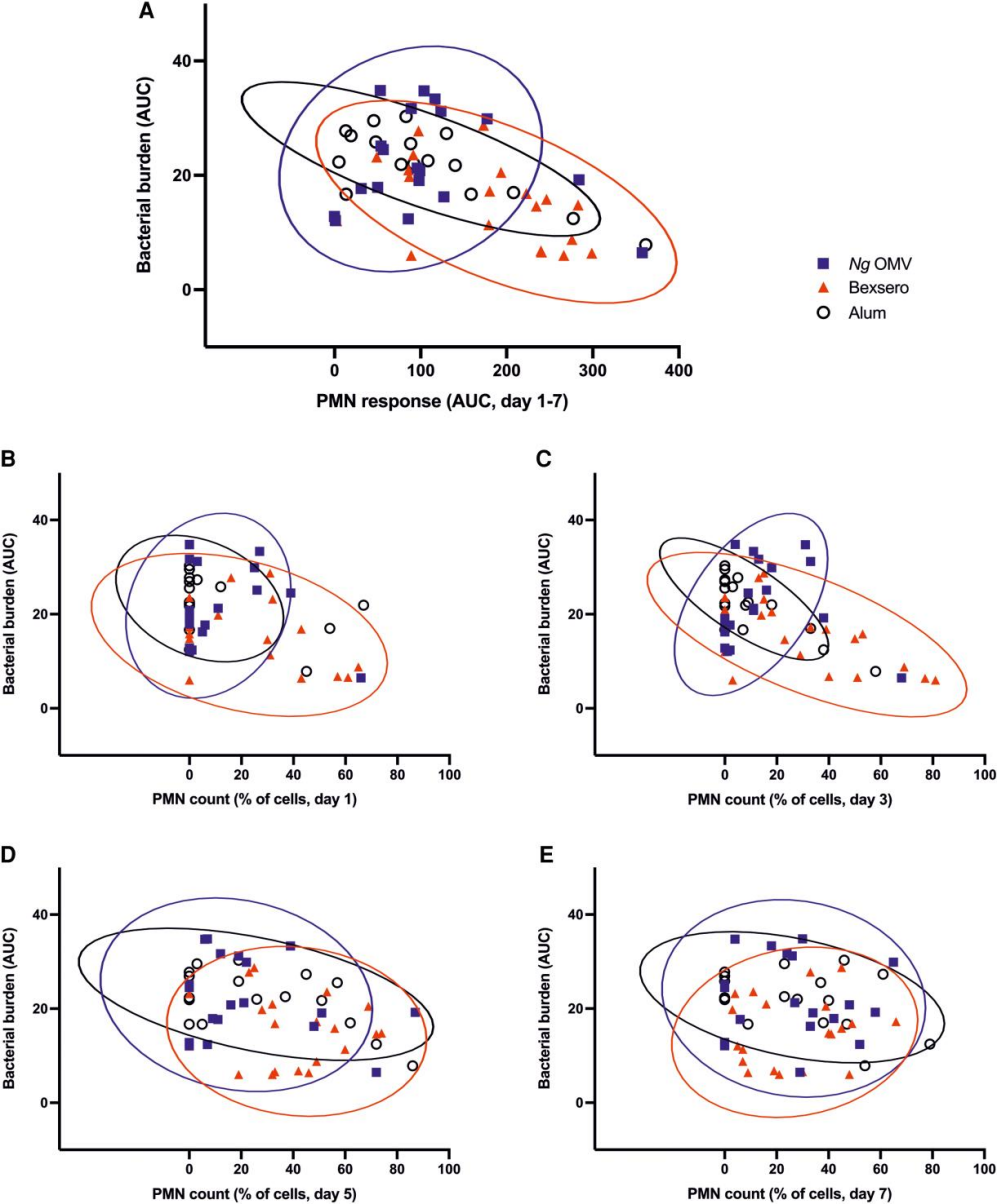
Correlation between PMN influx and bacterial burden in individual mice during challenge. PMNs were quantified as the percentage of 100 counted cells in stained vaginal smears. *A*, The AUC of PMN counts from days 1 to 7 was plotted against the AUC of the corresponding bacterial burden (in CFU/mL). Each point represents an individual mouse. PMN influx on days (*B*) 1, (*C*) 3, (*D*) 5, and (*E*) 7 was evaluated from mice immunized with *Ng* outer membrane vesicles (OMVs), Bexsero, or alum alone and plotted against the AUC of bacterial burden from the corresponding mouse. Ellipses are plotted at a 95% confidence level.

We also assessed PMN influx into the genital tract by measuring the percentage of PMNs among 100 cells in stained smears from vaginal swabs taken from mice vaccinated with Bexsero, *Ng* OMVs, or alum after challenge. The AUC for PMN counts from days 1–7 were calculated for each mouse and compared across immunization groups with bacterial burden during challenge (Figure 7*A*). Overall, an inverse correlation was observed between gonococcal burden and PMN influx, suggesting enhanced neutrophil recruitment is associated with improved control of infection. Pairwise differences between immunization groups were also assessed and revealed a significant difference between Bexsero and *Ng* OMV-vaccinated mice (Hotelling's T^2^ test, Bonferroni-adjusted *P* = .040). Interestingly, no differences were observed between *Ng* OMV and alum-only vaccinated mice (*P* = 1.000).

To assess the temporal dynamics of this effect, the same multivariate analysis was performed on PMN counts from each day and compared with bacterial burden. On day 3, Bexsero-vaccinated mice differed significantly from both *Ng* OMV-vaccinated (*P* = .046) and alum-vaccinated control mice (*P* = .041), while *Ng* OMV and alum control groups were indistinguishable (*P* = 1.000) (Figure 7*C*). The significant difference between Bexsero and *Ng* OMV was also observed on day 5 (*P* = .034) (Figure 7*D*), while no significant differences between groups were detected earlier (day 1, Figure 7*B*) or later (day 7, Figure 7*E*) after challenge.

Overall, Bexsero vaccination induced early PMN influx and reduced gonococcal burden, distinguishing it from *Ng* OMV and alum-only groups. The transient nature of these differences suggests that early neutrophil recruitment may contribute to enhanced bacterial clearance following Bexsero immunization.

## DISCUSSION

Understanding the immune mechanisms that correlate with, or mediate, protection following infection or vaccination is fundamental to vaccine design. Additionally, defining responses to OMV-based vaccines is complex due to their heterogeneous antigenic composition and by the limited protection offered by meningococcal OMVs against *Ng.*

Previous work defining correlates of protection examined responses conferred by meningococcal OMVs, identifying antigens by Western blot [15, 16]. Results implicated humoral and local cellular responses in protection, although the latter can only be assessed post-challenge, so may be confounded by *Ng* infection. We focused on characterizing IgG responses with protein antigen microarrays. We included mice immunized with *Ng* OMVs which were not protective, as predicted [17]. Integrating our results with data from vaccinees and infected individuals shows high concordance between murine and human IgG responses to Bexsero, supporting the utility of the murine model.

RmpM was omitted from *Ng* OMVs to avoid inducing blocking antibodies [29], while alum was used, similar to Bexsero. Zhu *et al* [16]. found that responses against a 37 kDa protein were associated with lower colonization levels of mice immunized with Bexsero; some proteins could fit this criterion (eg PorB, MetQ, NEIS0807, NEIS2653, NEIS2658, ComL) but we could not unambiguously identify this protein. *Ng* employs multiple immune evasion strategies [30], and vaccine preparations from unmodified gonococci may retain these properties so fail to induce protective immunity. Results from a Phase 1/2 study of gonococcal OMVs are awaited (https://clinicaltrials.gov/study/NCT05630859).

Our study differentiated the immunoprofiles elicited by Bexsero and *Ng* OMVs in serum and VW (Figures 3 and 4) which partly reflect antigen composition; strong anti-Opa responses in sera from animals vaccinated with *Ng* OMV likely reflect the extensive Opa protein repertoire in *Ng* [31]. It is likely that differences in responses to a combination of antigens, including MtrE and MetQ, which can induce enhanced clearance in the murine model [14], might be responsible for the reduced bacterial burden in Bexsero-immunized mice. Furthermore, although the OMVs in Bexsero have been detergent extracted [10], a significant proportion of IgG elicited by this vaccine in humans is directed at *Ng* lipooligosaccharide (LOS) [32]. Future work will define the profile of anti-LOS responses.

Bexsero and *Ng* OMVs both induced strong systemic and local IgG responses, with marked serum-VW correlation (Supplementary Figure 1), likely reflecting IgG transudation from serum to the mucosa. The lower IgA levels in VW are consistent with IgG predominating in the murine vaginal tract [33], with the neonatal Fc receptor, FcRn, responsible for transport of serum IgG into the female genital tract [34]. Future advances may enable accurate IgA quantification, which could be informative given its importance in mucosal clearance [35].

SBA titers differed significantly between Bexsero, *Ng* OMV and the alum groups (Figure 6*A*), but did not correlate with accelerated clearance (Figure 6*B*). Thus, SBA is an unreliable correlate of protection conferred by OMV vaccines against gonococcal mucosal infection, but might contribute to other vaccines [36, 37].

We employed a gradient boost methodology (XGBoost) to train our antigen microarray data against SBA and bacterial burden values from individual mice. We observed that PorB was consistently the most prominent antigen associated with SBA in linear models, in agreement with other recent studies, which also identified PorB as a target cross-strain bactericidal activity of monoclonal antibodies derived from Bexsero-immunized individuals [38]. Elevated anti-PorB responses in humans compared with mice might reflect boosting of memory responses established during carriage of *Neisseria* spp. [39]. Also, the absence of RmpM from *Ng* OMVs might promote PorB as a target for bactericidal antibodies [40]. In addition, we failed to find a strong correlation between IgG reactivities and vaginal bacterial burden. However, incorporating more data through further challenge experiments might provide sufficient statistical power to reliably identify antigens associated with protection.

Consistent with previous work [16], our findings indicate that Bexsero vaccination is associated with early PMN influx into the genital tract following challenge and accelerated bacterial clearance. *Ng* can resist killing within PMNs [41, 42]. However, Bexsero-induced antibodies or complement activation might render gonococci more susceptible to PMN-mediated elimination. Alternatively, the enhanced PMN influx may correlate with, but not mediate clearance.

Integrating murine data with data from a Bexsero clinical trial [12] showed striking similarity between mice and humans, suggesting that the patterns of antigen responses are similar in both species. These are well separated from the *Ng* OMV data. These findings highlight the clinical relevance of the murine model of gonococcal infection and are consistent with bacteria expressing immunosuppressive factors [30, 43].

In conclusion, our findings underscore the complexity of defining protective correlates against *Ng*, particularly with OMV-based vaccines. Although a recent policy offers Bexsero to adults at increased risk of sexually transmitted infection [44], randomized controlled trials in at-risk populations have failed to demonstrate protection [45]. Further randomized controlled trials with Bexsero in at-risk individuals are underway [21]. These trials combined with antigen microarray analysis could help dissect vaccine-induced immune responses. Despite these challenges, our results highlight the relevance of the murine model for pre-clinical evaluation and antigen discovery, and the promise of systems serology to extract immunological signals from complex datasets to accelerate design of effective gonococcal vaccines.

## Supplementary Material

jiag216_Supplementary_Data

## Supplementary Data

Supplementary materials are available at *The Journal of Infectious Diseases* online (http://jid.oxfordjournals.org/). Supplementary materials consist of data provided by the author that are published to benefit the reader. The posted materials are not copyedited. The contents of all supplementary data are the sole responsibility of the authors. Questions or messages regarding errors should be addressed to the author.
