# Supplementary material for: Investigation of Correlates of Protection Against Gonococcal Infection by Comparative Immunoprofiling of Responses in Experimental and Clinical Studies
Source: J Infect Dis. 2026 Apr 29;233(6):e1443–53. doi: 10.1093/infdis/jiag216 (PMC13271395; doi:10.1093/infdis/jiag216)
Supplement: jiag216_Supplementary_Data [file jiag216_supplementary_data.zip › Supplementary Table 1.docx]

| **Antigen** | **NEIS designation** | **NGO designation** |
| --- | --- | --- |
| Ape1 | NEIS1205 | NGO_0532 |
| BamA | NEIS0173 | NGO_1801 |
| BamD | NEIS0653 | NGO_0277 |
| BamE | NEIS0196 | NGO_1780 |
| CMP | NEIS0899 | NGO_1081 |
| ComP | NEIS1995 | NGO_1177 |
| FetA | NEIS1963 | NGO_2093 |
| fHbp | NEIS0349 | NGO_0033 |
| GNA1030 (NEIS1183) | NEIS1183 | NGO_0558 |
| GNA2091 (NEIS2071) | NEIS2071 | NGO_1985 |
| H.8 | NEIS1462 | NGO_0994 |
| IgA protease | NEIS0651 | NGO_0275 |
| LbpA | NEIS1468 |  |
| LbpB | NEIS1469 |  |
| Lipoprotein 1 | NEIS1063​ | NGO_0835​ |
| Lipoprotein 2 | NEIS0906​ | NGO_0948​ |
| LOLB | NEIS0814 | NGO_0439 |
| LptD | NEIS0275 | NGO_1715 |
| Maf1 | NEIS2083? | NGO_1972/NGO_1067 |
| Maf2 | NEIS1789 | NGO_1584/NGO_1393 |
| MAF2B |  | NGO_1068 |
| Membrane protein 1 | NEIS2704 | NGO_1559 |
| Membrane protein 2 | NEIS1304​ | NGO_0648​ |
| MetQ | NEIS1917 | NGO_2139 |
| MtrE | NEIS1632 | NGO_1363 |
| NEIS0729 | NEIS0729 | NGO_0358 |
| NEIS0807 | NEIS0807 | NGO_0166 |
| NEIS0807S | NEIS0807S | NGO_0432 |
| NEIS1066 | NEIS1066 | NGO_0834 |
| NEIS1125 | NEIS1125 | NGO_0783 |
| NEIS1164 | NEIS1164 | NGO_0690 |
| NEIS1253 | NEIS1253 | NGO_0588 |
| NEIS1287 | NEIS1287 | NGO_0666 |
| NEIS1404 | NEIS1404 | NGO_1040 |
| NEIS1407 | NEIS1407 | NGO_1044 |
| NEIS1474 | NEIS1474 | NGO_1215 |
| NEIS1487 | NEIS1487 | NGO_1225 |
| NEIS1632 | NEIS1632 | NGO_1363 |
| NEIS1853 | NEIS1853 | NGO_1683 |
| NEIS1947 | NEIS1947 | NGO_2109 |
| NEIS2075 | NEIS2075 | NGO_1981 |
| NEIS2647 | NEIS2647 | NGO_0554 |
| NEIS2653 | NEIS2653 | NGO_0694 |
| NEIS2658 | NEIS2658 | NGO_0751 |
| NEIS2666 | NEIS2666 | NGO_0891 |
| NEIS2687 | NEIS2687 | NGO_1155 |
| NEIS2697 | NEIS2697 | NGO_1430 |
| NEIS2698 | NEIS2698 | NGO_1431 |
| NEIS2724 | NEIS2724 | NGO_1847 |
| NEIS2733 | NEIS2733 | NGO_2086 |
| NEIS2779 | NEIS2779 | NGO_1276 |
| NGO2054 |  | NGO_2054 |
| NHBA | NEIS2109 | NGO_1958 |
| NspA | NEIS0612 | NGO_0233 |
| Opa1 |  | NGO_00350 |
| Opa10 | NEIS2198 | NGO_0868 |
| Opa2 |  |  |
| Opa3 |  | NGO_05420 |
| Opa4 |  | NGO_05620 |
| Opa5 |  | NGO_06725 |
| Opa58 |  | NGO_11100 |
| Opa6 |  | NGO_07725 |
| Opa7 |  | NGO_08230 |
| Opa8 |  | NGO_09965 |
| Opa9 |  | NGO_11100/NGO_0070 |
| OpaD |  | NGO_1513 |
| PAP2 | NEIS2626 | NGO_0499 |
| Phospholipase | NEIS1687 | NGO_1492 |
| PilC | NEIS0371 | NGO_0055 |
| PilE | NEIS0210 | NGO_10985 |
| PilN (PilP) | NEIS0411 | NGO_0097 |
| PilQ | NEIS0408 | NGO_0094 |
| PorB | NEIS2020 | NGO_1812 |
| PorB_1103_92 | NEIS2020_1103 | NGO_1812 |
| PorB_1140_93 | NEIS2020_1140 | NGO_1812 |
| PorB_513_87 | NEIS2020_513 | NGO_1812 |
| PorB_517_88 | NEIS2020_517 | NGO_1812 |
| PorB_537_89 | NEIS2020_537 | NGO_1812 |
| PorB_544_90 | NEIS2020_544 | NGO_1812 |
| PorB_699_91 | NEIS2020_699 | NGO_1812 |
| Potf3 | NEIS1689 | NGO_1494 |
| RmpM | NEIS1783 | NGO_1577 |
| Slam1 | NEIS1858 | NGO_1688 |
| SliC | NEIS1425​ | NGO_1063 |
| TamA | NEIS2112 | NGO_1956 |
| TamB | NEIS2113 | NGO_1955 |
| TbpA | NEIS1690 | NGO_1495 |
| TbpB | NEIS1691 | NGO_1496 |
| Ton1 | NEIS0338 | NGO_0021 |
| Ton2 | NEIS2646 | NGO_0553 |
| Ton3 | NEIS1428 | NGO_0952 |
| Ton4 | NEIS0944 | NGO_1205 |
| VacJ | NEIS1933 | NGO_2121 |
|  |  |  |

**Table S1 NGO And NEIS nomenclature for antigens in the microarray**
